# Endometriosis pain and epithelial neutrophil activating peptide-78 levels

**DOI:** 10.1038/s41598-022-07349-3

**Published:** 2022-02-25

**Authors:** Barbara Gardella, Mattia Dominoni, Andrea Gritti, Anna Arrigo, Silvia Antonucci, Giulia Vittoria Carletti, Valentina Musacchi, Giampiero Pietrocola

**Affiliations:** 1 Department of Clinical, Surgical, Diagnostic and Paediatric Sciences, University of Pavia, Pavia, Italy; 2 Department of Obstetrics and Gynecology, Fondazione IRCCS Policlinico San Matteo, Pavia, Italy; 3 Unit of Biochemistry, Department of Molecular Medicine, University of Pavia, Pavia, Italy

**Keywords:** Biochemistry, Cell biology, Chemical biology, Molecular biology

## Abstract

Endometriosis is a chronic gynecological disorder involved in the pathogenesis of chronic pelvic pain, based on a probable up regulation of the inflammatory system. The objective of the study is to investigate the peritoneal and serum levels of ENA-78 with the severity of endometriosis symptoms (dysmenorrhea, chronic pelvic pain and dyspareunia) using the visual analogue scale (VAS). This is a prospective case–control study that included 53 symptomatic women with evidence of endometriosis and 53 age-matched controls who underwent elective laparoscopic surgery for benign diseases. The concentration of ENA-78 was assessed in blood and peritoneal fluid samples in the follicular phase. In peritoneal fluid and plasma, the concentration of ENA-78 was significantly higher in cases than in controls (p < 0.001). A significant correlation was observed between peritoneal fluid ENA-78 levels and the severity of dysmenorrhea (Spearman Rho = 0.237; p = 0.014), and chronic pelvic pain (Spearman Rho = 0.220; p = 0.022) in endometriosis patients. Plasma levels ENA-78 showed a significant correlation with the severity (VAS score) of chronic pelvic pain (Spearman Rho = 0.270, p = 0.005 for cases), though a weak correlation was evident between plasma levels of ENA-78 and severity of dysmenorrhea (Spearman Rho = 0.083, p = 0.399 for cases). In conclusion, chronic pelvic pain in endometriosis is caused by changes of local and systemic activated chemokine patterns. These modifications involve the relationship between pro-inflammatory, angiogenic and angiostatic chemokines that modulate the severity of endometriosis associated symptoms.

## Introduction

Endometriosis is a chronic gynecological disorder, affecting at least 10% of reproductive-aged women and is characterized by the growth of endometrium‐like tissue outside the uterus. Ectopic endometrial cells in the peritoneal cavity are thought to cause pelvic pain and infertility. The presence of endometriotic lesion affects at least the immune and the reproductive systems^1–3^.

Several theories supported that a different immunological response facilitates the implants of endometrial cells in peritoneal cavity. Cytokines, chemokines, angiogenic factors, and growth factors are engaged in the proliferation and development of endometriotic lesions, as laboratory evidence has demonstrated^4–7^.

Although endometriosis may be asymptomatic, it can often cause debilitating pelvic pain, dyspareunia and dysmenorrhea or other symptoms including chronic fatigue^1^. The cause of the neuropathic endometriosis-related pain is unclear. The anatomical aspect of the lesions shows increased germination of Nerve Growth Factor (NGF)-mediated nerve fibers and an alteration of pro-inflammatory pattern, but the lack of correlation between severity of lesions and symptoms suggests the presence of other triggering factors^8^. The correlation between high levels of chemokines (chemotactic cytokines) and endometriosis was found for the first time in 1993^6^.

During the past 30 years, many researchers have evaluated different types of chemokines in patients with endometriosis in order to establish a potential role of chemokines in the pathogenesis of the disease^7^. Although the exact role of chemokines in the pathogenesis of endometriosis remains unsolved, it is clear that cytokines, chemokines, and angiogenic factors cause the development, progression and growth of ectopic endometrial tissue^9^ Furthermore, the chemokines could mediate and modulate the inflammatory response concerning endometriotic implants^9^. In particular, previous papers reported the association between Epithelial neutrophil-activating peptide-78 (ENA-78) expression and endometriosis neoangiogenesis. The chemokine ENA-78 is a neutrophil chemotactic factor, which activates neutrophils and promotes cytosolic- free calcium changes, and it induces neoangiogenesis^10,11^. ENA-78 is a C-X-C (cysteine separated by another amino-acid) of 8.3 KDa protein^10,11^. This chemokine is composed by 78 amino-acids and it contains four cysteines and it seems to be a homologue of IL-8^10,11^. The ENA-78 expression is induced by several inflammatory mediators and its production is stimulated by IL-1 and tumour necrosis factor-alpha (TNF-alpha) properly in stromal endometrial cells^12^. While the presence of cytokines, as IL-8 and IL-6, were predominantly expressed in glandular endometrial tissue, ENA-78 was expressed in the stromal component^10,11^. The high concentration of ENA-78, IL-6, and IL-8 were demonstrated in the peritoneal fluid of women with endometriosis, demonstrating their role in the pathogenesis of disease^13–16^. In addition, a recent study demonstrated that monocyte chemoattractant protein-1 (MCP-1), hepatocyte growth factor (HGF), and insulin-like growth factor-1 (IGF-1) in peritoneal fluid and serum contributed to the development of endometriosis^17^. Additionally the expression of vascular endothelial growth factor A, C-X-C-motif chemokine ligand 8, IL-6, and intercellular adhesion molecule-1 genes demonstrated an up-regulation in endometrial mesenchymal stem cells in cases of endometriois^16^. The peritoneal level of ENA-78 expression may be a reflection of the progression of endometriosis causing growth and maintenance of ectopic endometrial lesions, and stimulating leucocytes to produce growth factors and cytokines, and endometrial stromal proliferation with direct effects on these tissues^16,18^. The correlation between ectopic endometrial tissue and inflammatory status results in dysmenorrhea and chronic pain. For this reason in clinical practice, a visual analogue scale (VAS) may be used as a sensitive measure instrument of intensity and frequency of pelvic pain, which allows the determination of the characteristics of the symptoms from a patient’s perspective^19^.

The objective of the study is to investigate the peritoneal and serum levels of ENA-78 with severity of endometriosis symptoms (dysmenorrhea, chronic pelvic pain and dyspareunia) using the VAS scale.

## Materials and methods

### Subjects

This is a prospective case–control study carried-out at the Department of Obstetrics and Gynecology, IRCCS Policlinico S. Matteo Hospital, University of Pavia, Italy, between July 2014 to December 2018. The study included 53 symptomatic women with laparoscopic and histopathological evidence of endometriosis and 53 age-matched controls who underwent elective laparoscopic surgery for benign diseases or tubal sterilization. The inclusion criteria to be assigned as control were the following: (a) superimposable age (± 2 years) to index case; (b) no evidence of endometriosis during a careful abdominal and pelvic laparoscopic exploration performed immediately after each index case; (c) no history or evidence of pelvic inflammatory disease or malignancies: (d) no evidence of rheumatological or autoimmune disease. The study protocol was approved by the Hospital Ethical Committee (Comitato Etico of IRCCS Policlinico S. Matteo Hospital, protocol number 722-rcr2012-bis-23). The informed written consent was obtained from the patients. All methods and procedures were performed in accordance with the relevant guidelines and regulations.

All patients (control and study group) were recruited without hormonal intake and they underwent to surgical procedure in the follicular phase.

Both cases and controls received a preoperative routine assessment including pelvic examination and transvaginal ultrasonography. Carbohydrate Antigen 125 (CA-125) was measured in blood samples ^5mlofperipheralvenousblood^. The measure of CA-125 performed during the follicular phase at pre-operative assessment. Pelvic magnetic resonance imaging was performed only in three patients in which deep infiltrating endometriosis was suspected in order to assess the infiltration of the intestinal wall.

All cases had histological diagnosis of endometriosis on surgical biopsy. The patients with endometriosis were recruited for infertility, severe pelvic pain and suspected symptomatic deep endometriosis confirmed during the ultrasound exam. Both score and grade of endometriosis were evaluated during laparoscopy according to the revised criteria of the American Fertility Society (rAFS) (American Society for Reproductive Medicine, 1996)^10^.

Socio-demographic and clinical data were collected by an interview. Presence and severity of pain symptoms potentially related to the diagnosis were evaluated by administering a symptom-oriented questionnaire to each patient. Severity of symptoms (dysmenorrhea, chronic pelvic pain, dyspareunia, catamenial dyschezia/hematochezia, dysuria) was graded according to 10 items of the VAS scale (from 0 = no pain to 10 = unbearable pain). The impact of symptoms on sexual activity was scored according to the interference with sexual intercourse as follows: dyspareunia score 0 = absence of pain during intercourse; score 1 = mild dyspareunia, that does not interfere with the frequency of intercourse; score 2 = moderate dyspareunia, which reduces the frequency of intercourse; score 3 = severe dyspareunia, which disables intercourse. Blood sample and peritoneal fluids were collected from each patient on the day of surgery. All patients underwent surgery during the follicular phase of the menstrual cycle.

### Collection of plasma and peritoneal fluid

Blood samples were collected from an antecubital vein into EDTA tubes and then centrifuged at 1600 × *g* for 15 min to remove the cellular fraction. Plasma was immediately frozen at −80 °C in order to minimize protein degradation. Peritoneal fluid was collected at the beginning of each laparoscopy by using a cannula inserted in the pouch of Douglas, injecting 20 ml and subsequently drawing 10 ml of saline solution. The liquid withdrawn was centrifuged at 1600 × *g* for 15 min to separate the cellular fraction. The obtained supernatant was also immediately frozen at a −80 °C.

### Measurement of ENA-78 concentrations in plasma and peritoneal fluid

ENA-78 concentration was measured both in plasma and peritoneal fluid. Procarta Cytokine® Assay Kit (Affymetrix, Inc., Santa Clara, CA, USA) was used according to the manufacturer’s instructions. Briefly, after standard preparation, filter plate was wet with 150 µl/well of reading buffer and incubated for 5 min at room temperature. A mixture containing 5000 microspheres of ENA-78 was incubated with standards or samples in a final volume of 100 µl, for 30 min under continuous shaking. After three washes by vacuum filtration with washing buffer, diluted biotinylated antibodies against ENA-78 were added. After 30 min of incubation, Streptavidin-PE (Affymetrix, Inc. Santa Clara, CA, USA) diluted in assay buffer was added. At the end of 30 min of incubation, under continuous shaking and after washing, the fluorescence intensity of the beads was measured using the Luminex instrument, LabScan 100 (Luminex Corporation. Austin, USA) in a final volume of 120 µl of the assay buffer. Data analysis was performed with a Bio-Plex Manager software using a five-parametric logistic curve. The detection limit of the assay system for all antigens was 1 pg/ml.

### Statistical analysis

Statistical analysis was carried out with the Chi-square and Mann–Whitney U test to compare categorical and continuous variables, respectively. Correlations between severity of endometriosis-related symptoms as evaluated by VAS scales and concentrations of ENA-78 were evaluated by the Spearman rank correlation coefficient.

## Results

The case group had the following rAFS surgical evaluation: 16 women stage 1 (30.2%); 19 women stage 2 (35.9%); 7 women stage 3 (13.2%) and 11 women stage 4 (20.8%), respectively. In the control group, surgery confirmed benign conditions, including mature teratomas in 16 women (30.2%), cystoadenomas in 6 women (11.3%) and myomas in 18 women (34%). The remaining 13 control women (24.5%) were candidates for tubal sterilization.

Table 1 shows demographic and clinical data before the surgery. Plasma CA-125 concentration and rate of infertility were significantly higher in cases than in controls (p = 0.001 and p = 0.04, respectively). On the other hand, controls had higher BMI than cases (p = 0.03). Categorical analysis showed a high rate of dysmenorrhea and chronic pelvic pain among women with endometriosis as compared to controls (p < 0.001 and p = 0.001, respectively). Mean VAS scores for dysmenorrhea were significantly higher (p < 0.001) in cases (6.45 ± 2.85) than in controls (2.87 ± 3.47).

**Table 1:** Demographic characteristic of the patients considered in the study.

	Cases (n = 53)	Controls (n = 53)	p value
Age (years)	37 ± 7.56	36.8 ± 7.13	0.83
Body mass index (kg/m^2^)	21.71 ± 3.89	23.73 ± 5.18	0.03
CA 125 (U/ml)	45.74 ± 46.70	16.38 ± 15.06	**0.001**
**Previous pregnancy at term**			
0	38 (71.70%)	27 (50.94%)	0.07
1	8 (15.09%)	11 (20.76%)	
> 1	7 (13.21%)	15 (28.30%)	
**Infertility**			
No	39 (73.58%)	48 (90.57%)	0.04
Yes	14 (26.42%)	5 (9.43%)	
**Smoking**			
No	36 (67.93%)	43 (81.13%)	0.23
5–10 cigarettes/day	9 (16.98%)	4 (7.55%)	
> 10 cigarettes/day	6 (11.32%)	4 (7.55%)	
**Dysmenorrhea**			
Yes	48 (90.57%)	24 (45.29%)	** < 0.001**
No	5 (9.43%)	29 (54.71%)	
VAS (± SD)	6.45 ± 2.85	2.87 ± 3.47	** < 0.001**
**Chronic pelvic pain**			
Yes	24 (45.29%)	8 (15.10%)	**0.001**
No	29 (54.71%)	45 (84.90%)	
VAS (± SD)	5.60 ± 3.26	0.94 ± 2.30	** < 0.001**
**Dyspareunia**			
Yes	23 (43.40%)	14 (26.42%)	0.07
No	30 (56.60%)	39 (73.58%)	
VAS (± SD)	2.23 ± 2.91	1.68 ± 3.1	0.16
**Dyspareunia score**			
0	30 (56.60%)	39 (73.58%)	0.2
1	10 (18.87%)	4 (7.55%)	
2	11 (20.76%)	8 (15.10%)	
3	2 (3.77%)	2 (3.77%)	
**Catamenial dyschezia**			
Yes	10 (18.87%)	4 (7.55%)	0.15
No	43 (81.13%)	49 (92.45%)	
VAS (± SD)	1.15 ± 2.53	0.36 ± 1.15	0.085
**Catamenial hematochezia**			
Yes	2 (3.77%)	0 (0%)	0.49
No	51 (96.23%)	53 (100%)	
**Catamenial dysuria**			
Yes	1 (1.88%)	0 (0%)	1.0
No	52 (98.12%)	53 (100%)	

*VAS* visual analogue scale, average and standard deviation (SD).

Dyspareunia score 0 = absence of pain during intercourse; 1 = mild dyspareunia 2 = moderate dyspareunia; 3 = severe dyspareunia.

Mild chronic pelvic pain from at least 6 months was reported by 67.7% (11/17) of patients with stage I endometriosis; 73.7% (14/19) of patients with stage II suffered moderate chronic pelvic pain with VAS > 5, whereas 89% (16/18) of patients with stage III and IV reported severe pain (VAS > 8). As expected, 86.8% (46/53) of controls did not report chronic pelvic pain with an average VAS score of 0.94 ± 2.30, significantly lower compared with cases (5.60 ± 3.26; p < 0.001).

Mean VAS scores for dyspareunia (2.23 ± 2.91 in cases versus 1.68 ± 3.1 in controls, p = 0.16) and dyschezia (1.15 ± 2.53 in cases versus 0.36 ± 1.15 in controls; p = 0.085) did not differ between the two groups, likely due to the low percentage of nodules of the rectum-vaginal septum (3/53).

### Peritoneal fluid expression of ENA-78 in cases and controls

**Figure 1:**
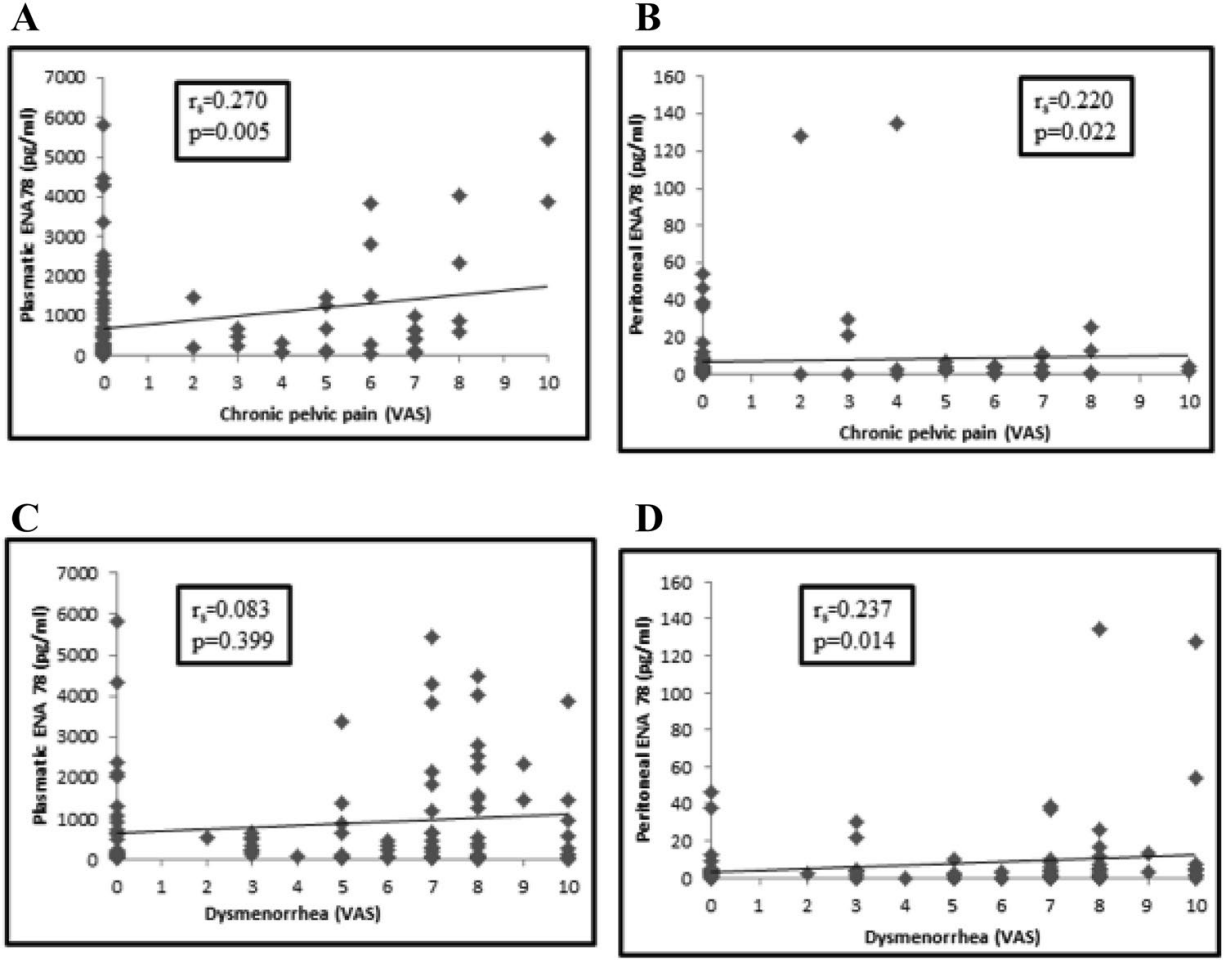
Correlation between the concentration of ENA 78 in plasmatic **(A)** and peritoneal **(B)** fluid and severity of chronic pelvic pain (VAS score). Correlation between the concentration of ENA 78 in plasmatic **(C)** and peritoneal **(D)** fluid and severity of dysmennorhea (VAS score). In the inset Sperman’s rank correlation coeficient (rs) and p value.

In peritoneal fluid, the concentration of ENA-78 was significantly higher in cases than in controls (12.4 ± 26.6 pg/ml for cases vs 2.2 ± 6.6 pg/ml; p < 0.001). In addition, a significant correlation was observed between peritoneal fluid ENA-78 levels and the severity of dysmenorrhea (Spearman Rho = 0.237; p = 0.014), and chronic pelvic pain (Spearman Rho = 0.220; p = 0.022) it is represented in Fig. 1.

**Figure 2:**
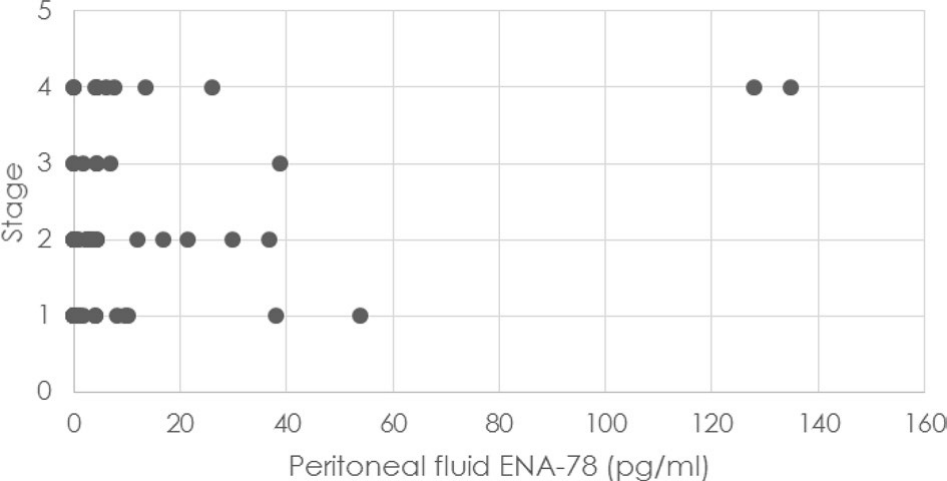
Correlation between the concentration of ENA-78 in peritoneal fluid and the stage of disease. In the inset Sperman’s rank correlation (r_s_ = 0.488)) coeficient and p value (p = 0.000).

Additionally, the ENA-78 level was higher in the peritoneal fluid in patients with endometriosis at stage IV than in the first stage of disease and its level increased in relation with different stages (Fig. 2).

### Plasma expression of ENA-78 in cases and controls

**Figure 3:**
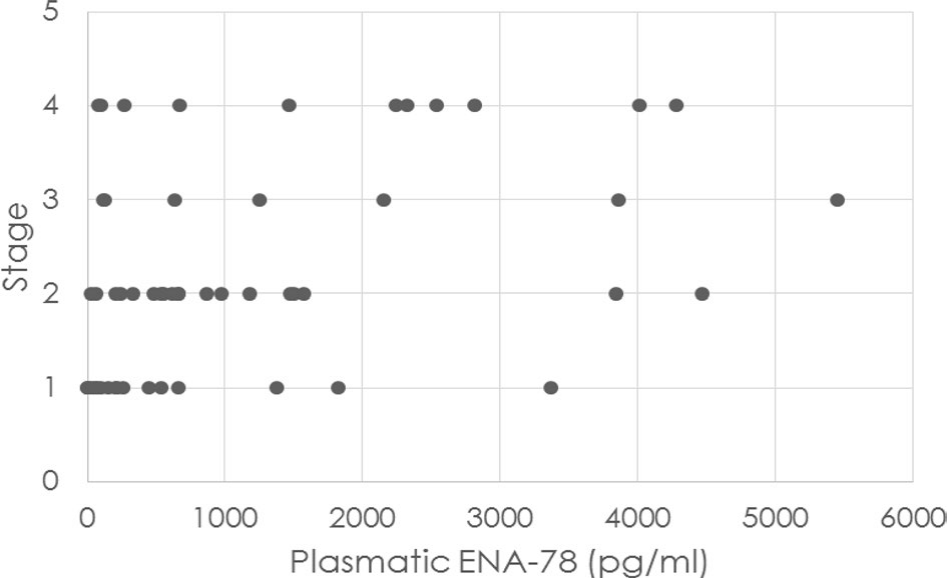
Correlation between the concentration of ENA-78 in plasmatic fluid and the stage of disease. In the inset Sperman’s rank correlation coeficient (r_s_ = 0.408) and p value (p = 0.000).

Plasma levels of ENA-78 was significantly higher in women with endometriosis than in controls (p < 0.001, 1260.4 ± 1391.8 pg/ml for cases vs 508.5 ± 1066.1 pg/ml for controls). Interestingly, ENA-78 showed a significant correlation with the severity (VAS score) of chronic pelvic pain (Spearman Rho = 0.270, p = 0.005 for cases) (Fig. 1). However, a weak correlation was evident between plasma levels of ENA-78 and the severity of dysmenorrhea (Spearman Rho = 0.083, p 0.399 for cases), as it is represented in Fig. 1. The plasma level of ENA-78 was higher in stage IV endometriosis than in the first stage of disease, and its level increased in relation with different stages and the severity of endometriosis, as it is represented in Fig. 3.

## Discussion

ENA-78 has an important role in the pathogenesis of rheumatoid arthritis, gastric and inflammatory bowel diseases, ovarian carcinoma, psychiatric syndromes (infant autism, depression), chronic prostatitis, and gastric and lung cancer^11,20–24^. In fact, ENA-78 contains ERL element (Glu-Leu-Arg) that can cause neutrophil chemotaxis, cytosolic-free calcium changes, and angiogenesis in inflammatory tissue, but it is less active in the release of neutrophil granulocytes^11,25^, macrophages, fibroblasts, endothelial cells, monocytes, epithelial tissue, platelets, alveolar and intestinal epithelial cells producing ENA-78: this process is involved in the recruitment of neutrophils and mononuclear cells in the sites of inflammatory processes and in the production of molecules involved in leukocyte adhesion. This mechanism is able to increase the production of inflammatory cytokines^13,26–28^. Literature data has previously reported that the expression of ENA-78 by endometrial stromal cells were significantly stimulated by lipopolysaccharide (LPS), Il-1beta and TNF-alpha in peritoneal fluid of women with endometriosis^11^.

It is probable that the ectopic endometrial tissue can induce an inflammatory pattern in the site of its implantation with a rise in levels of cytokines which lead to an increase in the secretion of ENA-78^12,16,29–32^. In addition, the macrophage activation induced by ENA-78 can increase the recruitment of peritoneal lymphocytes responsible for chronic local inflammation and amplify the expression of multiple pro-inflammatory mediators of pain^33,34^. For example, in ectopic endometriotic lesions there is a direct relationship between the number of macrophages and the density of nerve fibers, suggesting a direct relationship between immune activation and generation of pain^35^. In a recent study also the adipose tissue, in the retroperitoneal cavity adjacent to pelvic endometriosis, was more fibrotic and showed signs of neo-angiogenesis with a significantly higher level of infiltration of macrophages in the endometriosis group^36^.

In addition, endometriosis pain is probably not only caused by the local immune response since it is well known that not in all cases, the removal of lesions improves pain relief (20–28% of patients do not have pain relief after surgery)^37,38^.

Probably the proinflammatory systemic response could explain the sensory nerve activation and altered nociceptive pathways^39–41^.

Moreover we found that also the plasma concentration of ENA-78 is higher in the patients with endometriosis than in controls and is directly correlated to the severity of pain, especially to chronic pelvic pain and dysmenorrhea as a possible expression of systemic immune activation.

This inflammatory response is probably able to sustain the continuous proliferation of cytokines and chemokines and the consequent abnormal response to pain stimulus. The finding of increased plasma concentrations of ENA-78 in patients with endometriosis compared to controls and the direct correlation between plasma ENA-78 and the severity of chronic pelvic pain suggest that additional pathways other than local mechanisms, can regulate endometriosis-related pain.

Peripheral sensitization causes the drop of threshold for neuronal activation, inducing pain from a stimulus that does not normally provoke pain (allodynia) or heightens existing pain (hyperalgesia)^42^. If inflammation persists, nociceptors can become chronically hypersensitive even after inflammation resolution. This peripheral hypersensitivity of nociceptive fibers could explain allodynia and hyperalgesia of endometriosis^39^.

This theory is supported by animal models in which an up-regulation of CXCR2 and CXCR3 receptors and their chemokine ligands exist in populations of neurons in chronic pain models^16^.

Furthermore, we can speculate that the altered pattern of chemokine activity causes hyper-excitability at when regarding the level of down-regulation of neurons that causes a central sensitization that maintains chronic pelvic pain even in the absence of lesions^43^.

In conclusion, our study suggests that chronic pelvic pain in endometriosis is associated with changes of local and systemic chemokine patterns. These modifications involve the relationships between pro-inflammatory, angiogenic and angiostatic chemokines that modulate the severity of associated symptoms.

Since the up-regulation of chemokines and their receptors could be one of the mechanisms that contributes to the severity of chronic pelvic pain, these molecules may represent a specific therapeutic target. In fact, the efficacy of currently used anti-inflammatory drugs and hormonal therapy is limited^44^. New therapeutic approaches to block the effects of the cytokine cascade could be a promising way for the treatment of patients with symptomatic endometriotic lesions non-responsive to conventional therapy and could have a benefit from the development of new immunomodulatory drugs.
